# Anti-*Helicobacter pylori* activity and Structure-Activity Relationship study of 2-Alkylthio-5-(nitroaryl)-1,3,4-thiadiazole Derivatives

**Published:** 2013

**Authors:** Ali Asadipour, Najmeh Edraki, Maryam Nakhjiri, Azadeh Yahya-Meymandi, Eskandar Alipour, Parastoo Saniee, Farideh Siavoshi, Abbas Shafiee, Alireza Foroumadi

**Affiliations:** a Department of Medicinal Chemistry, Faculty of Pharmacy and Neuroscience Research Center, Kerman University of Medical Sciences, Kerman, Iran.; b Department of Medicinal Chemistry, Faculty of Pharmacy and Pharmaceutical Sciences Research Center, Tehran University of Medical Sciences, Tehran, Iran.; c Medicinal and Natural Products Chemistry Research Center, Shiraz University of Medical Sciences, Shiraz 71345, Iran.; d Department of Chemistry, North Tehran Branch, Islamic Azad University, Tehran, Iran.; e Department of Microbiology, Faculty of Sciences, University of Tehran, Iran.*.*

**Keywords:** 1, 3, 4-thiadiazole, anti-*Helicobacter pylori *activity, Nitroheterocycle

## Abstract

Nitro-containing heteroaromatic derivatives structurally related to nitroimidazole (Metronidazole) are being extensively evaluated against *Helicobacter pylori* isolates. On the other hand, 1,3,4-thiadiazole derivatives have also demonstrated promising antibacterial potential. In present study, we evaluated anti-*H. pylori *activity of novel hybrid molecules bearing nitroaryl and 1,3,4-thiadiazole moieties.

Anti-*H. pylori* activity of novel 5-(5-nitroaryl)-1,3,4-thiadiazole derivatives bearing different bulky alkylthio side chains at C-2 position of thiadiazole ring, were assessed against three different metronidazole resistant *H. pylori *isolates by paper disk diffusion method.

Most of the compounds demonstrated moderate to strong inhibitory response especially at 25 μg/disk. The structure-activity relationship study of the compounds demonstrated that introduction of different alkylthio moieties at C-2 position of thiadiazole ring; alter the inhibitory activity which is mainly dependent on the type of C-5 attached nitrohetercyclic ring. The promising compound of this scaffold, bearing 1-methyl-5-nitroimidazole moiety at C-5 and α-methylbenzylthio side chain at C-2 position of thiadiazole ring, showed strong inhibitory response against metronidazole resistant *H. pylori* isolates at 12.5 μg/disk (the inhibition zone diameter at all evaluated concentrations (12.5- 100 μg/disk) is >50 mm).

Novel 5-(5-nitroaryl)-1,3,4-thiadiazole scaffold bearing different C-2 attached thio-pendant moieties with promising anti-*H. pylori* potential were identified. Among different nitroheterocycles, 5-nitrofuran and 5-nitroimidazole moieties were preferable for the substitution at C-5 position of 1,3,4-thiadiazole ring. Introduction of different alkylthio side chains at C-2 position of central ring alter the inhibitory activity which is mainly dependent on the type of C-5 attached nitrohetercyclic ring.

## Introduction

*Helicobacter pylori *(*H. pylori*), an invading gram negative microaerophilic bacterium causes common chronic gastric infection known as the major cause of gastric and gastrodudenal ulcer disease (1-2). In addition, chronic infection of *H. pylori *is attributed to adenocarcinoma and low grade B-cell mucosa-associated lymphoid tissue lymphoma of stomach and therefore; it is the first bacterium known as definite carcinogen by the World Health Organization’s International Agency for Research on Cancer (3-5). The treatment of *H. pylori *is complicated and includes combination of proton-pump inhibitor (PPI) agents with two or more different antibiotics such as amoxicillin, clarithromycin, metronidazole, tetracycline, and levofloxacin for at least 7 days (2, 6). Although eradication of infection results in ulcer healing and reduces the risk of gastric cancer (7), however; emerging resistance to current antibiotics is encountered as a major concern in the treatment of *H. pylori *infection (8). From this, different factors such as poor patient compliance, common side effects of combination therapy and also significant cost of multi-drug therapy, limits the eradication of infection and therefore reveals the urgent need to search novel candidates for the eradication or prevention of infection (7-8). 

Recent studies reported some novel natural sources (9-13) and synthetic scaffolds (14-15) as the therapeutic agents with significant inhibitory activity against *H. pylori *isolates. Among the different chemical structures, nitro-containing heteroaromatic derivatives (such as nitrofuran and nitrothiophen derivatives) that are structurally related to nitroimidazole family (Metronidazole) are being extensively evaluated against *Helicobacter pylori *isolates (16). In this regard, the current nitroheterocyclic antibiotics, tinidazole and furazolidone have demonstrated promising anti-*H. pylori *properties to be used in place of metronidazole (17). The results of different studies indicate that the possible mechanism of antimicrobial activity of 5-nitoheterocycles involves the reduction of the 5-nitro group with the formation of reactive intermediates that can interact to various cell macromolecules. Therefore; the nitohetrocycle moiety is essential for the antimicrobial activity of these derivatives (18). 

**Figure 1:**
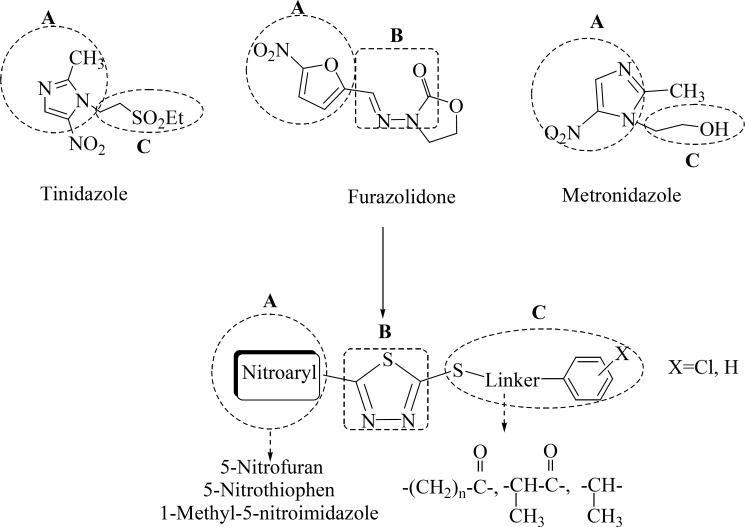
Chemical structure of 2-alkylthio-5-(nitroaryl)-1,3,4-thiadiazoles (compounds 1-13) derived from the structure of current anti-*H. pylori *nitroheterocyclic agents (tinidazole, furazolidone and metronidazole).

On the other hand, the antibacterial property of 1,3,4-thiadiazole derivatives against both Gram(+) and Gram(-) bacteria has been well established (19-21). We have previously demonstrated the anti-*Helicobacter pylori *properties of different 5-(5-nitroaryl)-1,3,4-thiadiazoles with different substitutes at C-2 position of thiadiazole ring. The structure-activity relationship study of these compounds indicates that the anti-*H. pylori *property of these scaffolds is highly dependent on the type of C-2 substitute. Different C-2 attached moieties to 1,3,4-thiadiazole derivatives such as piperazine derivatives and different sulfur containing side chains were evaluated (22-24). In continuation of our research program on 2,5-disubstituted-1,3,4-thiadiazole scaffold (20-25), we have previously reported novel 2-substituted-thio-1,3,4-thiadiazoles containing different 5-nitroaryl substitutes on C-5 position of thiadiazole ring with potent antilishimanial properties (26). These compounds could also be served as potent scaffold against *H. pylori *infection: (Figure 1)

1- The nitroaryl ring (A) comprises 5-nitrofuran (which is derived from the nitroaromatic part of furazolidone), 5-nitrothiophen and 1-methyl-5-nitroimidazol substitutes (that are isosterically replaced instead of nitroaromatic part of furazolidone and metronidazole; respectively). 

2- The 1,3,4-thiadiazole ring (B) could partly mimic the methyleneamino-oxazolidinone fragment of furazolidone and enhances the antibacterial and particularly anti-*H. pylori *properties of scaffold. 

3- Substituted-thio side chain (C) of these novel derivatives could serve as a new part against the resistant strains of *H. pylori *and affects the potency of these derivatives against the resistant isolates of this organism.

We have previously reported the synthetic pathway of designed compounds “1-13**”**. The chemical structure of these compounds is demonstrated in Table 1. The structure of these compounds was confirmed by elemental analysis, ^1^H NMR and IR spectra (26).

According to our ongoing research program on 1,3,4-thiadiazole derivatives as potent antiparasitic scaffold (20-26), herein, we report the anti-*H. pylori *activity of 2-alkylthio-5-(nitroaryl)-1,3,4-thiadiazole derivatives.

**Table 1:** Average of inhibition zone diameters of compounds "1-13" at different doses against three metronidazole resistant *H. pylori *isolates

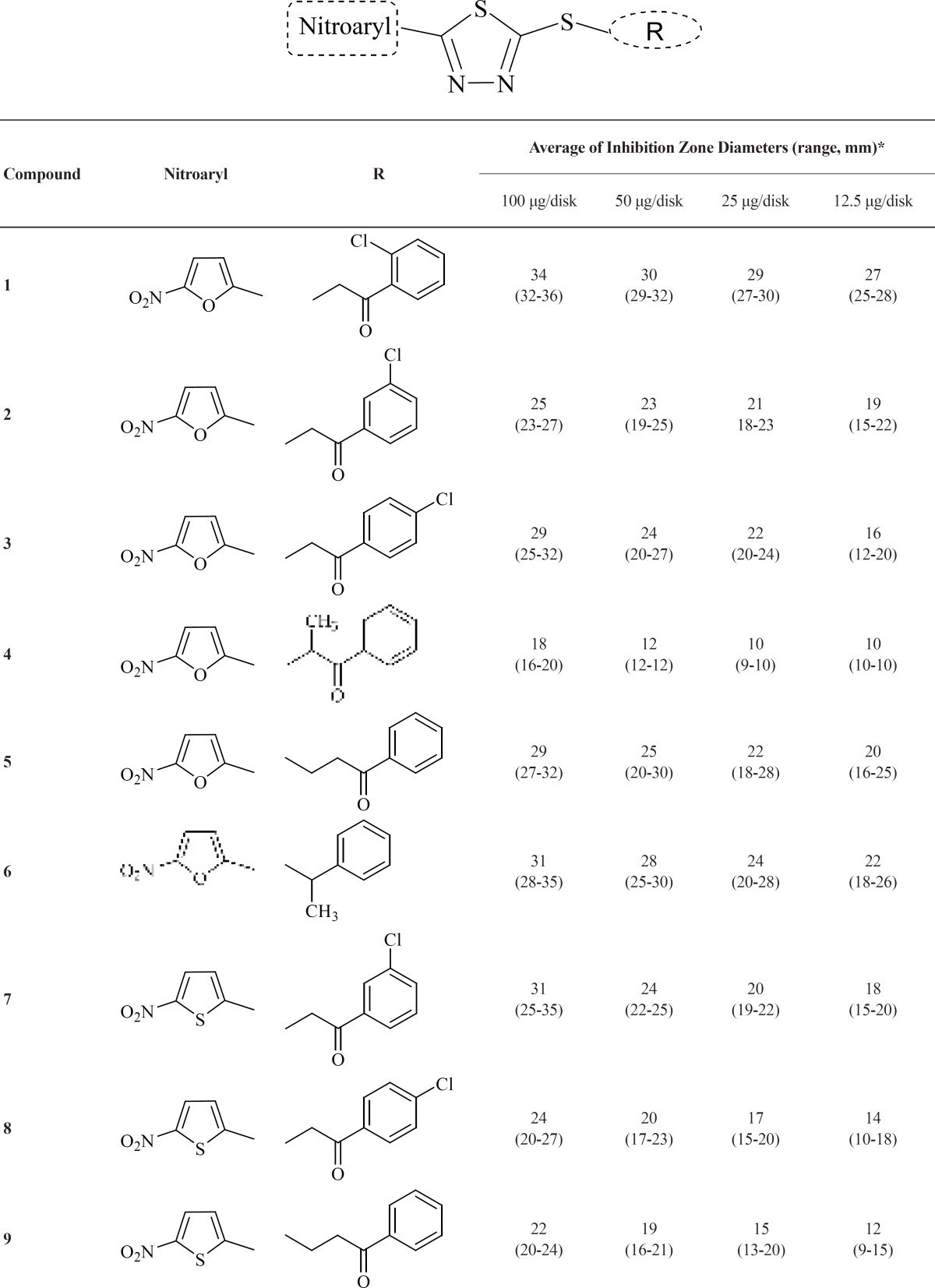

* The anti-*Helicobacter pylori *activity was determined by the paper disc diffusion bioassay

## Experimental

*Bacterial isolates and culture conditions*

Clinical *H. pylori *isolates from gastric biopsy specimens were obtained from Shariati Hospital (Tehran, Iran). Primary isolation was performed on a selective blood agar base (Oxoid, Basingstoke, UK) supplemented with horse blood (5%, v/v) and 1 Selectatab tablet (500 mg; Mast Diagnostics, Merseyside, UK). Following primary selective isolation, *H. pylori *bacteria cells were identified according to colony morphology, Gram staining, microaerophilic growth at 37 °C, oxidase +, catalase +, urease +, nitrate -, H_2_S, and hippurate hydrolysis. Growth of *H. pylori *was maintained at 37 °C for 3-5 days in an atmosphere of 5% O_2_, 15% CO_2_, and 80% N_2_ in an anaerobic chamber (Hirayama, Tokyo, Japan). To maintain a moist atmosphere, a moist paper towel was placed in the chamber. Bacterial strains were stored at -70 °C in brain heart infusion broth (BHIB) (Difco, East Molesey, UK) containing 10% (v/v) fetal calf serum (FCS) and 15% (v/v) glycerol. Frozen clinical isolates were thawed and inoculated on Mueller-Hinton agar (MHA) plates (Oxoid), supplemented with 10% horse blood and incubated under microaerophilic conditions. Given the importance of inoculum homogeneity, cellular viability was controlled microscopically by morphological observation with Gram staining. In order to control the proportions of coccoid cells in the cultures, cultures were always used after 48 h of incubation, when they generally did not present coccoid forms. Bacterial growth was taken from the plates and resuspended in sterile saline. The inoculum was prepared to contain 5 *× *10^7^ CFU/mL by adjusting the turbidity of the suspension to match the McFarland No. 2 standard.

*Bacterial growth inhibition assay (disk diffusion method)*

Growth inhibition was performed by the filter paper disk diffusion method on selective Brucella agar with 7% defibrinated horse blood under microaerophilic conditions at 37 °C. The samples were evaluated for their anti-*Helicobacter *activity, dissolved in dimethylsulfoxide (DMSO). All compounds were assayed against metronidazole-resistant *H. pylori *strains at 3 concentrations (100, 50, and 25 and 12.5 μg/disk); the surfaces of the *Brucella* blood agar plates were inoculated with 100 μL of bacterial suspensions. Blank standard disks (6 mm in diameter) were deposited on the plates and impregnated with 10 μL of different dilutions of test compounds. Following incubation for 3-5 days at 37 °C, the inhibition zone around each disk (average diameter), if any, was recorded. The control disks received 10 μL of DMSO. All tests were performed in triplicate and the antibacterial activity was expressed as the mean of inhibition diameters (mm) produced by the tested compounds. 

The one-way analysis of variance (ANOVA) followed by Tukey multiple comparisons was used to analyze the data. A value of p < 0.05 was considered as the significance level between the groups.

## Result and discussion

The anti-*Helicobacter pylori *activity of novel 1,3,4-thiadiazole derivatives “1-13**” **was determined by paper disk diffusion bioassay against three metronidazole resistant *H. pylori *isolates. The average of inhibition zone diameters of compounds in three isolates at different doses (100, 50, 25 and 12.5 μg/ disk) is summarized in Table 1. The anti-*H. pylori *activity was classified as follows: strong response, zones range diameter *> *20mm; moderate response, zone diameter 16-20 mm; weak response, zone diameter 11-15 mm; and little or no response, zone diameter *< *10 mm (22).

*Structure activity relationship*

*Assessment of 5-nitroaryl substitute*

The inhibition zone diameter (IZD) of test compounds indicates that most of the compounds exhibited strong anti-*H. pylori *at concentration of 100-25 μg/disk against metronidazole resistant strains. All the test compounds could be classified into three groups according to the 5-nitroaryl substitute: 5- nitrofuran “1-6”, 5-nitrothiophen “7-10” and 1-methyl-5-nitroimidazol (nitroimidazole) “11-13” derivatives. The results demonstrated that compound 13, containing 1-methyl-5- nitroimidazol substitute at C-5 position and α-methylbenzylthio-pendant group at C-2 position of the 1,3,4-thiadiazole ring, is the promising compound of these series with the most potent anti-*H. pylori *activity (the inhibition zone diameter of this compound at all evaluated concentrations (12.5-100 μg/ disk) is > 50 mm). In general; comparison of the inhibition zone diameter of compounds at different doses revealed that most of the nitroimidazole and nitrofuran derivatives demonstrated strong anti-*H. pylori *response at 25 μg/disk (IZD > 20mm); while the nitrothiophen derivatives (compounds “**7- **10”) showed moderate (IZD:16-20 mm) to weak (IZD:11-15 mm) response at 25 μg/disk. For instance, the average of inhibition zone diameter of 5-nitrofuran and 5-nitroimidazole derivatives containing *α*-methylbenzylthio-pendant substitute at C-2 position of central ring (compounds 6 and 13) at 25 μg/disk was 24 mm and > 50mm respectively. While the 5-nitrothiophen counterpart (compound 10) demonstrated weak response at this dose (IZD: 12 mm) [F(6, 10) = 8.640, p = 0.042 (p < 0.05)]. Therefore; it seems that the preferable nitroaryl substitution at C-5 position of 1,3,4-thiadiazole ring is as follows. 

5-nitroimidazol>5-nitrofuran> 5-nitrothiophen 

*Assessment of 2-alkylthio side chain*

Different C-2 attached substituted-thio side chains were evaluated in 5-nitroaryl-1,3,4 thiadiazole derivatives against metronidazole resistant *H. pylori *isolates (Table 1). Regio-isomeric chlorine substitution and *α*-methylphenacylthio-pendant group were investigated. Comparison of different derivatives reveals that the inhibitory effect of alkylthio side chain is partly dependent on the type of nitroheterocyclic moiety. Different regio-isomer of chlorine in phenacylthio side chain demonstrated strong inhibitory response especially in 5-nitrofuran (compounds 1-3) and 5-nitroimidazole derivatives (compounds 11- 12) at concentration range of 100-25 μg/disk. Among the nitrothiophene derivatives, meta-chlorine substituted phenacyl thio (compound 7) demonstrated strong inhibitory response at the concentration range of 100-25 μg/disk. 

Among the nitorofuran derivatives, ortho-chlorine substituted phenacylthio and *α*-methylbenzylthio-side chain (compounds 1 and 6; respectively), demonstrated the strong inhibitory potential especially at 12.5 μg/disk against the resistant strain. In addition, *α*-methyl branching of phenacylthio-side chain (compound 4) diminishes the inhibitory response; the corresponding inhibition zone diameter at 50- 12.5 μg/disk was 10-12 mm [F(1,4) = 9.16, p = 0.014 (p < 0.05)]. 

The best inhibitory response in 5-nitroimidazole series could also be achieved by the introduction of *α*-methylbenzylthio-side chain; compound 13 with inhibition zone diameter of > 50 at all the tested concentrations. While introduction of this side chain in 5-nitrothiophen derivatives, generates compound with weak anti-*H. pylori *activity at all concentrations (compound 10).

## Conclusion

In conclusion, we have identified novel 2-*α*Alkylthio-5-(nitroaryl)-1,3,4-thiadiazole derivatives with promising anti-*H. pylori* potential. Among different nitroaryls, 5-nitrofuran and 5-nitroimidazole moieties are preferable for substitution at C-5 position of 1,3,4-thiadiazole ring. Introduction of different substituted-thio side chains at C-2 position of central ring alter the inhibitory activity which is mainly dependent on the type of C-5 attached nitrohetercyclic ring. 
